# Donor lymphocyte infusions for recurrence of myeloid neoplasms after allogeneic hematopoietic cell transplantation in the era of hypomethylating agents and BCL2 inhibitors

**DOI:** 10.1007/s00277-026-06973-7

**Published:** 2026-03-30

**Authors:** Miriam Mozaffari Jovein, Thomas Meyer, Miguel Waterhouse, Dietmar Pfeifer, Jesús Duque-Afonso, Michael Lübbert, Kristina Maas-Bauer, Ralph Wäsch, Hartmut Bertz, Justus Duyster, Robert Zeiser, Jürgen Finke, Claudia Wehr

**Affiliations:** 1 Department of Medicine I, Faculty of Medicine, Medical Center - University of Freiburg, Freiburg, Germany; 2 Collaborative Research Institute Intelligent Oncology (CRIION), Freiburg, Germany; 3 German Cancer Consortium (DKTK), Freiburg, and German Cancer Research Center (DKFZ), Heidelberg, Germany; 4 Signalling Research Centres BIOSS and CIBSS - Centre for Integrative Biological Signalling Studies, University of Freiburg, Freiburg, Germany

**Keywords:** Allogeneic stem cell transplantation, Relapsed AML, Myeloid neoplasms, Hypomethylating agents, Donor lymphocyte infusions, Venetoclax

## Abstract

The combination of hypomethylating agents (HMA) and venetoclax (VEN) has transformed acute myeloid leukemia (AML) treatment. Data on donor lymphocyte infusion (DLI) with HMA/VEN for relapse after allogeneic hematopoietic cell transplantation (alloHCT) remain limited.

We retrospectively analyzed 78 adults with relapsed myeloid neoplasms after first alloHCT between 2018 and 2025. DLI was given with HMA, HMA/VEN, or other/no treatments. The primary endpoint was event-free survival (EFS), defined as time to death or second alloHCT.

Median time to relapse after alloHCT was 12.9 months (range 2.2-192.4). Initial DLI doses ranged from 0.3 to 8.14 × 10⁶ CD3⁺ cells/kg. Median EFS after first DLI was 15.2 months: with 16.2 (DLI/HMA), 14.3 (DLI/HMA/VEN), and 21.1 (DLI/other), respectively. Death occurred in 25.6% and second alloHCT in 32.1% of patients. GvHD of any kind after DLI treatment manifested in 30.8%, both events comparable across groups. Complete remission (CR) after DLI was achieved in 42.3% after a median of 4.2 months, including patients with *TP53* mutations (*n* = 9).

Approximately 40% of patients with relapsed myeloid malignancies were successfully salvaged with DLI and combination treatments. Patients with high-risk features such as morphological relapse were overrepresented in the DLI/HMA/VEN cohort but achieved comparable outcomes to the other treatment groups. This finding suggests successful treatment of even morphological relapse by addition of VEN to DLI/HMA regimens while supporting the need for controlled trials.

**Supplementary Information:**

The online version contains supplementary material available at 10.1007/s00277-026-06973-7.

## Introduction

Allogeneic hematopoietic cell transplantation (alloHCT) is the standard therapy for high risk and relapsed myeloid neoplasms [1]. However, progression of the underlying disease or overt relapse after alloHCT is frequent and progression of the primary disease is the leading cause of mortality in patients over three months after alloHCT [2]. Donor lymphocyte infusions (DLI) enhance inherent graft-versus-leukemia (GvL) activity and offer an impactful therapeutic approach either to prevent or treat relapse after alloHCT [3–11]. DLI treatment for relapse after alloHCT has been reported to achieve comparable overall survival rates as second alloHCT [12].

To enhance their clinical efficiency, DLI are often combined with additional treatments such as chemotherapy, interferons, FLT3 inhibitors, BCL-2 inhibitors or DNA hypomethylating agents (HMA) [13–19]. In a post-alloHCT setting, administration of HMA augments GvL effect by inducing higher CD8 + T-cell responses to tumor antigens, offering a treatment alternative in elderly patients unfit for chemotherapy or second alloHCT [20–22].

In recent years, the combination of HMA and the BCL-2 inhibitor venetoclax (VEN) has expanded the therapeutic options for patients diagnosed with myeloid neoplasms [17, 23–26]. However, data on effectiveness of combinations of DLI and HMA/VEN treatment in post-alloHCT settings is scarce [27]. In our study, we aimed to investigate the clinical response as well as adverse events of patients receiving DLI combined with HMA+/-VEN in case of relapsed myeloid neoplasms after alloHCT.

## Patients and methods

### Patient inclusion criteria and data collection

Patients with myeloid malignancies who experienced molecular (increasing molecular markers, increase/new onset of mixed chimerism) or hematological relapse after alloHCT and received DLI-treatment after December 31st 2017 were included (*n* = 78), excluding patient data previously published in our centers last DLI-follow-up study [28]. Conditioning intensity was determined using the transplant conditioning intensity (TCI) score as previously described [29, 30]. Data was collected prospectively as part of standard clinical care and database management and analyzed retrospectively. Follow-up data lock was June 4th 2025. All analyses were performed in accordance with the Declaration of Helsinki and approved by the local Ethics Committee (22-1490-S1-retro, 24-1470-S1). All patients provided written consent for the use of their data for clinical research.

### DLI and concomitant treatments

DLI were either stored aliquots of the G-CSF mobilized peripheral blood stem cell graft obtained prior to alloHCT or acquired by repeated peripheral, unmobilized mononuclear cell harvest from the donor post-alloHCT. DLI aliquots were cryopreserved in 97.2% of applications. DLI treatment protocols followed established guidelines as well as institutional standards after individual risk evaluation [7]. Performance status, previous record of graft-versus-host disease (GvHD), donor match and current GvHD status were taken into consideration when deciding on DLI doses and intervals. The number of total DLI cycles per patient and the length of DLI treatment were determined individually by tolerance and disease control as well as DLI availability.

**Fig. 1:**
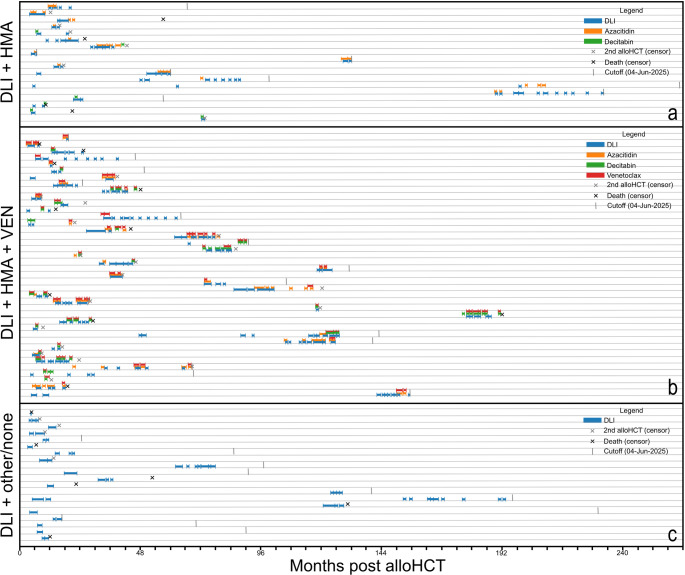
Treatment timelines of individual patients. The panels show individual treatment timelines from HCT until censoring or death for (**a**) patients receiving DLI/HMA (n=18), (**b**) patients receiving DLI/HMA/VEN (n=40) and (**c**) patients receiving DLI/other or no treatment (n=20). DLI, donor lymphocyte infusion; HMA, hypomethylating agent; HCT, hematopoietic cell transplantation; VEN, venetoclax

We stratified the cohort according to accompanying DLI treatment into three groups (Fig. 1): (1) DLI/HMA (*n* = 18), (2) DLI/HMA/VEN (*n* = 40) and (3) DLI/other or no treatment (*n* = 20). Patients receiving HMA/VEN at any timepoint were included in the DLI/HMA/VEN group, except for one patient only ever receiving one single dosage of VEN before discontinuation of VEN treatment due to intolerance/hypersensitivity. HMA+/-VEN treatment followed institutional standard protocols: HMA treatment consisted of either azacitidine 100 mg/m²/d day 1–5 (standard protocol in combination with DLI at our institution, equaling a cumulative dose of 500 mg/m², comparable to the 75 mg/m²/day day 1–7 protocol used without DLI) or decitabine 20 mg/m²/d day 1–5. VEN was administered on days 1–7 during HMA/VEN + DLI cycles. All patients were initiated with full-dose VEN treatment (100 mg/d with azole or 400 mg/d without azole). Depending on baseline leucocyte count and tumor lysis syndrome risk, step-up dosing was performed during the first cycle, ranging from 50 to 100 mg/d (with azole) or 100-400 mg/d (without azole) until reaching full dose. In selected patients with advanced hematological relapse and high disease burden, initial VEN treatment was extended up to 28 days for disease cytoreduction prior to switching to standard DLI dosing protocols. In subsequent cycles, VEN was mainly disrupted or dose-reduced due to infections or neutropenia (Supplementary Fig. 4). Once remission was achieved, VEN treatment was discontinued and subsequent cycles consisted of HMA + DLI or DLI monotherapy. Similarly, in the DLI/HMA cohort, HMA was typically discontinued upon achievement of remission. Treatment discontinuation or modification also occurred due to adverse events, particularly infections (Supplementary Fig. 4) and hematological toxicity. Any other treatments and patients without accompanying treatments were summarized in the DLI/other or no treatment cohort for statistical significance. “Other” treatments included intensive chemotherapy (CPX-351, polychemotherapy), PD1-inhibitors (nivolumab, pembrolizumab), targeted therapy (dasatinib, imatinib, midostaurine, gilteritinib, ruxolitinib), surgery and irradiation of extramedullary disease, hydroxyurea as well as PEG-Interferon either as a single drug or in various combination therapies.

### Response evaluation and mutation analysis

CR for AML was defined in accordance with current international guidelines [1]. For MDS and MPN, CR was adapted to the post-alloHCT setting: Given that traditional hematological response criteria are often confounded by transplant-related factors, CR in MDS and MPN patients required normalization or stabilization of blood counts in the absence of transfusion dependence, combined with complete donor chimerism and clearance of molecular markers when applicable. These criteria were applied in accordance with the principles outlined in international MDS and MPN response guidelines while accounting for the unique challenges of response assessment in the post-transplant context [31, 32]. Individual minimal residual disease (MRD) markers were selected prior to alloHCT using pre-transplant blood and bone marrow samples following best available knowledge at the time of transplant. For mutation analysis an Illumina TrueSight^®^ Myeloid NGS panel was used, which allows to screen for mutations in 54 genes and hotspots. Intronic or silent variants, as well as known single nucleotide polymorphisms (SNPs) were excluded. Limit of detection was in general 3% at primary diagnosis and 1% at relapse.

After alloHCT, peripheral blood count, bone marrow blood count and minimal residual disease markers were monitored according to institutional routine follow-up protocols for early relapse detection. This included routine bone-marrow and peripheral blood follow-up at day + 30, day + 100 and day + 365 after alloHCT. Chimerism analysis was assessed by fluorescence in situ hybridization (FISH) or by short tandem repeat (STR) analysis and INS/DEL bei digital PCR [33]. Mixed chimerism (MC, > 1%) was considered an additional risk factor in the prediction of molecular or hematological relapse. MRD monitoring methods and laboratory analyses were updated according to evolving standards. Disease risk classification and MRD monitoring were aligned with the current recommendations from the European LeukemiaNet (ELN) [1]. Hematological relapse was defined according to ELN MRD guidelines as either ≥ 5% blasts in bone marrow or circulating blasts in peripheral blood, molecular relapse was defined as a significant increase of MRD markers without an increase in peripheral or bone marrow blasts during follow-up analysis [34]. For MDS and MPN, hematological relapse was defined as new or worsening cytopenia and constitutional symptoms in combination with mixed chimerism and/or increasing molecular markers, as to account for the multiple reasons for cytopenia post-alloHCT while considering pre-alloHCT recommendations for response evaluation in the respective diseases [31, 32]. Additional relapse signs were manifestations of extramedullary disease.

### Data analysis and statistics

Data was analyzed using R (Boston, Massachusetts, US) version 4.3.2, Microsoft Office Excel (Redmond, Washington, US) version 2410 and Python (Python Software Foundation, Wilmington, Delaware, USA) version 3.11.6. Figures were designed using R (Boston, Massachusetts, US) version 4.3.2, Python (Python Software Foundation, Wilmington, Delaware, USA) version 3.11.6 and Affinity Designer 2 (Nottingham, ENG) version 2.5.6.

Primary endpoint of our analysis was EFS after first DLI dosage with an event being defined as either death or consecutive alloHCT. Further data of interest were overall survival (OS), time from alloHCT to relapse or to first DLI dosage, as well as patient and treatment specific characteristics. Survival estimates were made using Kaplan Meier curves. Curve comparison was performed using log-rank test. Results were expressed as hazard ratios (HR) with 95% confidence intervals (95% CI). *p* < 0.05 was considered as statistically significant. Baseline characteristics and event-to-event times were summarized using median and range for continuous measures, for cumulative DLI graft size comparison, mean values and range were calculated.

For visualization of therapy timelines (Fig. 1), we extracted therapy dates (DLI, azacitidine, decitabine, venetoclax) from clinical notes using automated text analysis with four different language processing algorithms (gpt-oss-120b, llama scout, mistralai_Mistral-Small-3.1-24B, and Qwen2.5-72B-Instruct) to ensure accuracy. Dates were converted to months after first alloHCT. A treatment episode was recorded only when at least two algorithms independently identified the same therapy date, reducing the risk of extraction errors. Treatment windows were defined as 30-day periods and extended when additional treatment dates were identified within or adjacent to existing windows. Follow-up was censored at second alloHCT, death, or the administrative data cutoff (June 4, 2025), whichever occurred first.

## Results

### (Pre-) alloHCT characteristics of the cohort

We included 78 patients with myeloid neoplasms who received alloHCT between 11/1999 and 06/2019 and experienced relapse between 01/2018 and 06/2025 (Table 1; Fig. 1). Median age at alloHCT was 58.6 years (range 22.2–75.5 years) and evenly distributed in all cohorts. De novo acute myeloid leukemia (AML) was diagnosed in 53.8% of patients, secondary AML (sAML) in 16.7%, myelodysplastic neoplasia (MDS) in 11.5%, myeloproliferative neoplasm (MPN) in 7.7%, treatment-related AML (tAML) in 9.0% and one case of treatment-related MDS (tMDS, 1.3%). Adverse risk AML was most prevalent in the DLI/HMA/VEN cohort: 57.5% (*n* = 23) compared to DLI/HMA: 38.9.3% (*n* = 7) and DLI/other or no treatment: 25.0% (*n* = 5) (Table 1). Pre-transplant remission was CR in 23.1% of patients and active disease in 76.9% of patients (DLI/HMA: 61.1%, DLI/HMA/VEN: 85.0%, DLI/targeted/none: 75.0%). The median time from diagnosis to alloHCT was 3.9 months (range 0.2–80.4). GvHD prophylaxis included backbones of cyclosporine A (CsA) and mycophenolate (MPA) in 83.3% of cases and was conducted with serotherapy in 79.5% of cases. Graft source was mobilized stem cells collected in peripheral blood by leukapheresis in most of the cases (Table 1).

**Table 1:** Patients, HCT and treatment characteristics

	Entire cohort	DLI/HMA	DLI/HMA/VEN	DLI/other or no treatment
Number of patients	78	18	40	20
Age at HCT in years (median, range)	58.6 (22.2-75.5)	60.6 (52.6-75.4)	57.8 (22.2-73.2)	60.0 (30.9-75.5)
Sex				
female, *n* (%)	26 (33.3%)	5 (27.5%)	15 (37.5%)	6 (30.0%)
male, *n* (%)	52 (66.6%)	13 (72.2%)	25 (62.5%)	14 (70.0%)
Time from diagnosis to HCT (months), median (range)	3.9 (0.2-80.4)	3.5 (0.7-53.6)	3.5 (0.2-14.4)	5.2 (1.4-80.4)
Disease				
de novo AML, *n* (%)	42 (53.8%)	11 (61.1%)	22 (55.0%)	9 (45.0%)
sAML, *n *(%)	13 (16.7%)	3 (16.7%)	5 (12.5%)	5 (25.0%)
tAML, *n* (%)	7 (9.0%)	0 (0.0%)	6 (15.0%)	1 (5.0%)
MDS, *n* (%)	9 (11.5%)	2 (11.1%)	6 (15.0%)	1 (5.0%)
tMDS, *n* (%)	1 (1.3%)	1 (5.6%)	0 (0.0%)	0 (0.0%)
MPN, *n* (%)	6 (7.7%)	1 (5.6%)	1 (2.5%)	4 (20.0%)
Molecular risk group in AML				
favorable, *n* (%)	8 (10.3%)	3 (16.7%)	2 (5.0%)	3 (15.0%)
intermediate, *n* (%)	21 (26.9%)	5 (27.8%)	9 (22.5%)	7 (35.0%)
adverse, *n* (%)	35 (44.9%)	7 (38.9%)	23 (57.5%)	5 (25.0%)
n.a., *n* (%)	14 (17.9%)	3 (16.7%)	6 (15.0%)	5 (25.0%)
IPSS-M risk group in MDS/tMDS				
very low	0 (0.0%)	0 (0.0%)	0 (0.0%)	0 (0.0%)
low	3 (3.8%)	2 (11.1%)	1 (2.5%)	0 (0.0%)
moderate low	2 (2.6%)	0 (0.0%)	2 (5.0%)	0 (0.0%)
moderate high	1 (12.8%)	0 (0.0%)	0 (0.0%)	1 (5.0%)
high	1 (12.8%)	0 (0.0%)	1 (2.5%)	0 (0.0%)
very high	3 (3.8%)	1 (5.6%)	2 (5.0%)	0 (0.0%)
Remission prior to first HCT				
CR, *n* (%)	18 (23.1%)	7 (38.9%)	6 (15.0%)	5 (25.0%)
Active disease, *n* (%)	60 (76.9%)	11 (61.1%)	34 (85.0%)	15 (75.0%)
Donor match				
MRD, *n* (%)	16 (12.8%)	3 (16.7%)	6 (15.0%)	7 (35.0%)
MUD, *n* (%)	47 (60.3%)	9 (50.0%)	31 (77.5%)	7 (35.0%)
MMRD, *n* (%)	3 (3.8%)	1 (5.6%)	1 (2.5%)	1 (5.0%)
MMUD, *n* (%)	12 (15.4%)	5 (27.8%)	2 (5.0%)	5 (25.0%)
Donor sex				
female, *n* (%)	21 (26.9%)	3 (16.6%)	13 (32.5%)	5 (25.0%)
male, *n* (%)	57 (73.1%)	15 (83.4%)	27 (67.5%)	15 (75.0%)
Donor age (years), median (range)	30 (19-65)	31 (20-52)	28 (19-65)	44 (20-65)
Conditioning therapy				
MAC, *n* (%)	13 (16.7%)	1 (5.6%)	8 (20.0%)	4 (20.0%)
BU/Cy based, *n* (%)	2 (15.4%)	0 (0.0%)	2 (25.0%)	0 (0.0%)
TT/BU/FLU*, *n *(%)	11 (84.6%)	1 (100.0%)	6 (75.0%)	4 (100.0%)
Toxicity-reduced, *n* (%)	65 (83.3%)	17 (94.4%)	32 (80.0%)	16 (80.0%)
FLU/BCNU/MEL, *n* (%)	16 (24.6%)	5 (29.4%)	7 (21.9%)	4 (25.0%)
FLU/TT-based **, *n* (%)	49 (75.4%)	12 (70.6%)	25 (78.1%)	12 (75.0%)
Graft source				
PBSC, *n* (%)	77 (98.7%)	17 (94.6%)	40 (100.0%)	20 (100.0%)
BM, *n* (%)	1 (1.3%)	1 (5.6%)	0 (0.0%)	0 (0.0%)
CD34+ cells*10^6/kg bw, median (range)	6.2	6,055	6.41	6.3
GvHD prophylaxis				
Backbone CsA+MPA ***, *n* (%)	65 (83.3%)	15 (83.3%)	34 (85.0%)	16 (80.0%)
With serotherapy (ATLG or alemtuzumab), *n* (%)	62 (79.5%)	14 (77.8%)	34 (85.0%)	14 (70.0%)
no serotherapy ***, *n* (%)	16 (20.5%)	4 (22.2%)	6 (15.0%)	6 (30.0%)
Overall prevalence of GvHD pre-DLI, *n* (%)	40 (51.3%)	8 (44.4%)	20 (50.0%)	12 (60.0%)
aGvHD pre-DLI, *n* (%)	35 (44.9%)	7 (38.9%)	17 (42.5%)	11 (55.0%)
aGvHD grade I-IV, *n* (%)	20/8/5/2 (58.9/22.9/14.3/5.7%)	5/2/0/0 (71.4/28.6/-/-%)	10/3/3/1 (58.8/1.8/1.8/5.9%)	5/3/2/1 (45.5/27.3/18.2/9.1%)
cGvHD pre-DLI, *n* (%)	6 (7.7%)	1 (5.6%)	4 (10.0%)	1 (5.0%)
cGvHD stage mild/moderate/severe, *n* (%)	3/2/1 (50.0/33.3/16.7%)	0/0/1 (-/-/100%)	2/2/0 (50.0/50.0/-%)	1/0/0 (100/-/-%)
Time from first HCT to first hematological/molecular relapse (months), median (range)	12.9 (2.2-192.4)	16.9 (2.9-192.4)	14.8 (2.2-172.3)	10.2 (3.0-188.9)
Patients still under immunsoppression at timepoint of relapse, *n* (%)	10 (12.8%)	2 (11.1%)	4 (10.0%)	4 (20.0%)

Shown are transplant specific as well as pretreatment specific characteristics for the entire cohort (*n*=78) as well as for the subgroups according to accompanying treatment under DLI treatment (DLI/HMA, *n*=18; DLI/HMA/VEN, *n*=40; DLI/other or no treatment, *n*=20)

* includes one patient with FLU/BU

** includes FLU/TT/MEL, TT/FLU/Tre, TT/FLU/TreRIC

*** single patients receiving everolimus instead of CsA: 3 patients receiving Certican/MPA/ATLG, 1 patient receiving Certican mono, 1 patient receiving Certican/MPA, 1 patient receiving Certican/MPA/Cyclophosphamide. 4 of the Certican patients are included in the DLI/HMA/VEN cohort, while 1 patient receiving Certican is included in the DLI/HMA and DLI/other or no treatment cohort, respectively

*HCT* hematopoietic cell transplantation, *DLI* donor lymphocyte infusion, *HMA* hypomethylating agent, *VEN* venetoclax, *AML* acute myeloid leukemia, *sAML* secondary AML, *tAML* therapy-associated AML, *MDS* myelodysplastic syndrome, *tMDS* therapy-associated MDS, *MPN* myeloproliferative neoplasm, *CR* complete remission, *MRD* matched related donor, *MUD* matched unrelated donor, *MMRD* mismatched related donor, *MMUD* mismatched unrelated donor, *MAC* myeloablative conditioning, *BU* busulfane, *Cy* cyclophosphamide, *TT* thiotepa, *FLU* fludarabine, *BCNU* carmustine, *MEL* melphalane, *Tre* Treosulfane, *RIC* reduced intensity conditioning, *PBSC* peripheral blood stem cells, *BM* bone marrow, *kg* kilogram, *bw* body weight, *GvHD* graft versus host disease, *CsA* cyclosporine A, *MPA* mycophenolic acid, *ATG* anti-thymocyte globuline, *PT-Cy* post-transplantation cyclophosphamide, *MTX* methotrexate, *aGvHD* acute GvHD, *cGvHD* chronic GvHD, *n.a.* not assessed, *FU* follow-up, *EFS* event-free survival

### Relapse after alloHCT and DLI

Time from alloHCT to first relapse was 12.9 months in median (range 2.2-192.4) in the entire cohort and shortest in patients receiving DLI/other or no treatment (median 10.2 months, range 3.0-188.9; Table 1, Supplementary Fig. 1). Time from alloHCT to first DLI was shortest in the DLI/other or no treatment cohort with a median of 8.9 months (range: 3.2-121.9 months), contrasting a median of 12.6 months (range 3.0-186.2 months) in the entire cohort, 12.0 months (range: 3.8-186.2 months) in the DLI/HMA, and 14.8 months (range 3.0-174.8 months) in the DLI/HMA/VEN cohort (Table 2). Hematological relapse triggered treatment in 50.0% of cases and was the most common trigger for treatment in the DLI/HMA/VEN cohort (33.3% of DLI/HMA, 62.5% of DLI/HMA/VEN and 40.0% DLI/other or no treatment; Table 2).

**Table 2:** DLI treatment characteristics

	Entire cohort	DLI/HMA	DLI/HMA/VEN	DLI/other or no treatment
Number of patients	78	18	40	20
Total DLI applications	473	102	263	108
Number of DLI doses per patient, median (range)	5 (1-21)	4.5 (2-13)	6 (1-18)	4 (1-21)
Initial trigger for DLI treatment				
Molecular/cytogenetic relapse or mixed chimerism, n (%)	39 (50.0%)	12 (66.7%)	15 (37.5%)	12 (60.0%)
Hematological relapse, n (%)	39 (50.0%)	6 (33.3%)	25 (62.5%)	8 (40.0%)
Extramedullary, n (%)	3 (7.7%)	0 (0.0%)	2 (7.7%)	1 (14.3%)
Time from HCT to 1st DLI (months), median (range)	12.6 (3.0-186.2)	12 (3.8-186.2)	14.8 (3.0-174.8)	8.9 (3.2-121.9)
Time from stopp immunosuppression to 1st DLI (months), median (range)	6.7 (0.1-174.3)	5.0 (0.1-174.3)	8.6 (0.2-171.6)	4.5 (0.2-115.0)
Patients receiving DLI while still under immunosuppression, n (%)	3 (3.8%)	2 (11.1%)	0 (0.0%)	1 (5.0%)
Intervalls between DLI doses (days), median (range)	29 (3.1-4242.6)	30.5 (6.1-4084)	35 (3.1-3942.7)	28 (9.2-4242.6)
Time from 1st to 2nd DLI dose (days), median (range)	25.9 (15.3-442.3)	24.4 (6.1-119)	27.5 (15.3-442.3)	21.4 (12.2-36.6)
Time from 1st to last DLI dose (days), median (range)	177 (18.3-5715.7)	114 (18.3-5715.7)	297 (21.4-4498.8)	105.4 (21.4-5621.2)
Cumulative DLI cell count (x106 CD3+/kgbw), mean (range)	21.11 (0.56-101.82)	14.57 (2.29-51.58)	26.94 (0.56-101.82)	14.71 (1.56-94.87)
Overall prevalence of GvHD after DLI, n (%)	24 (30.8%)	5 (27.8%)	13 (32.5%)	6 (30.0%)
aGvHD, n (%)	14 (17.9%)	1 (5.6%)	8 (20.0%)	5 (25.0%)
Grade I/II/III/IV, n (%)	6/5/3/0 (42.9/35.7/21.4/-%)	1/0/0/0 (100/-/-/-%)	3/3/2/0 (37.5/37.5/25.0/-%)	2/2/1/0 (40.0/40.0/20.0/-%)
cGvHD, n (%)	13 (16.7%)	5 (27.8%)	5 (12.5%)	4 (20.0%)
mild/moderate/severe, n (%)	5/2/6 (38.5/15.4/46.2%)	1/2/2 (20/40.0/40.0%)	3/1/1 (60.0/20/20%)	1/0/3 (25.0/0/75.0%)
Best response after DLI				
CR, n (%)	33 (42.3%)	6 (33.3%)	17 (42.5%)	10 (50.0%)
PERS/SD, n (%)	18 (23.1%)	4 (22.2%)	10 (25.0%)	4 (20.0%)
PD, n (%)	20 (25.6%)	6 (33.3%)	10 (25.0%)	4 (20.0%)
n.a., n (%)	7 (9.0%)	2 (11.1%)	3 (7.5%)	2 (10.0%)

Shown are treatment characteristics under DLI treatment for the entire cohort (*n*=53, column B) as well as for the subgroups according to accompanying treatment under DLI treatment (DLI/HMA, *n*=18; DLI/HMA/VEN, *n*=40; DLI/other or no treatment, *n*=20)

*Several patients included in NIFAR study (Nivolumab) before or after HMA+/-Venetoclax treatment, single patient combined with local irradiation of extramedullary manifestation

**Treatment for patients receiving neither HMA mono nor HMA/VEN prior to DLI consists of CLAM, Gilteritinib, Sorafenib+Midostaurin/Gilteritinib+Midostaurin, Glivec/Dasatinib/Syrea, Jakavi, PEG-Interferon

*HCT* hematopoietic cell transplantation, *DLI* donor lymphocyte infusion, *HMA* hypomethylating agent, *VEN* venetoclax, *n.a.* not assessed, *kg* kilogram, *bw* body weight, *OS* overall survival, *aGvHD* acute graft versus host disease, *cGvHD* chronic graft versus host disease, *CR* complete remission, *PERS* persisting disease, *SD* stable disease, *PD* progressive Disease, *CLAM* clorafabine, cytarabine and mtoxantrone, *Glivec* Imatinib, Syrea, hydroxycarbamide, *Jakavi, ruxolitinib* PEG-interferon, polyethylene glycol interferon

**Fig. 2:**
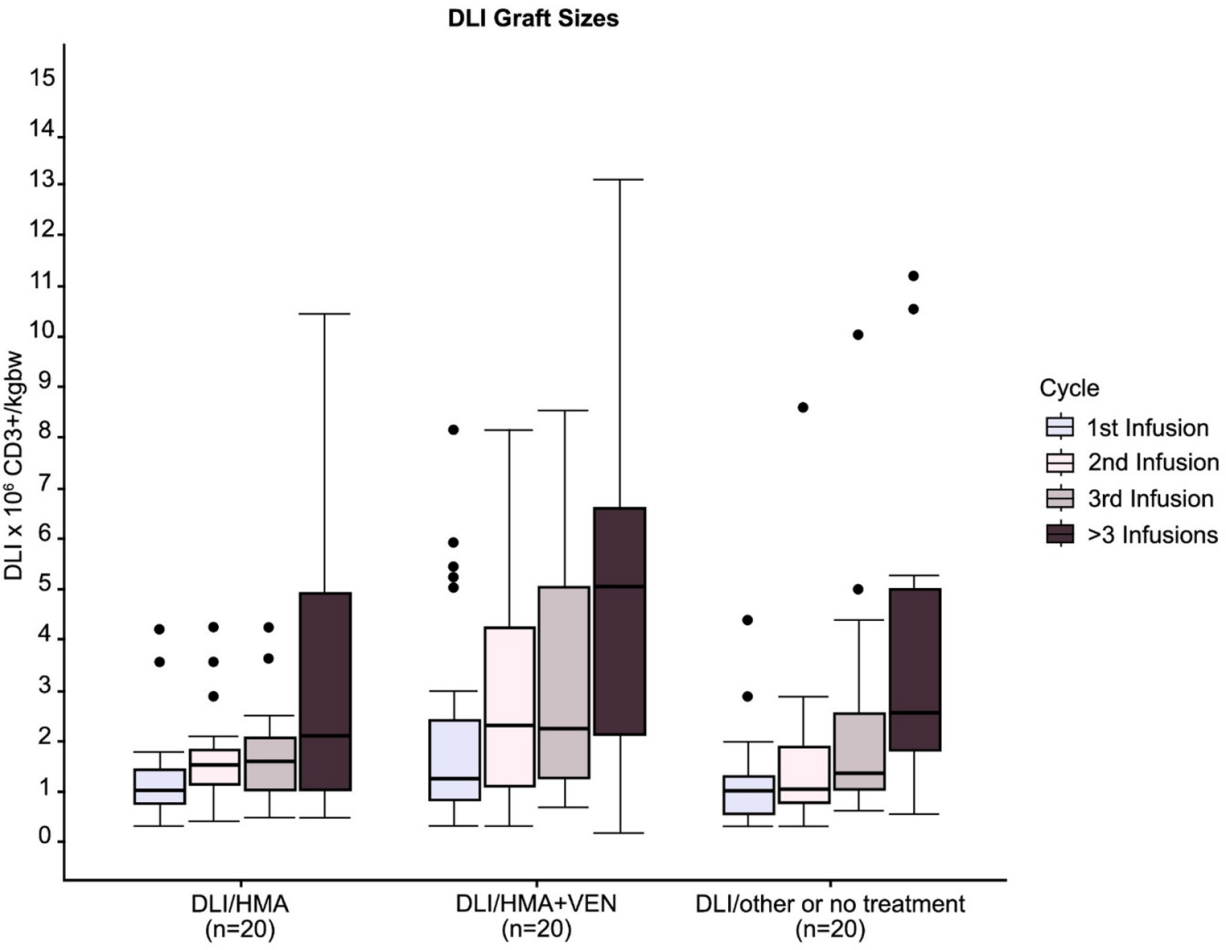
DLI graft sizes. Shown are DLI graft sizes for first, second, third and following DLI treatments in 106 CD3+ cells/kg bodyweight. Graft size comparisons are shown for patients receiving DLI/HMA (left group, *n*=18), patients receiving DLI/HMA/VEN (middle group, *n*=40) and patients receiving DLI/other or no treatment (right group, *n*=20). DLI, donor lymphocyte infusion; CD3, cluster of differentiation 3; HMA, hypomethylating agent; HCT, hematopoietic cell transplantation; kg, kilogram; bw, body weight, VEN, venetoclax

Initial DLI graft sizes consisted of 0.3–8.14 × 10^6^ CD3^+^ cells/kgbw throughout the entire cohort and had the biggest range in the DLI/HMA/VEN cohort (median 1st DLI counts: HMA 0.31–4.20 × 10^6^ CD3^+^ cells/kgbw, HMA/DLI/VEN 0.3–8.14 × 10^6^ CD3^+^ cells/kgbw, DLI/other or no treatment 0.32–4.39 × 10^6^ CD3^+^ cells/kgbw). Step-up graft counts as well as mean cumulative DLI graft cell count were also highest in the DLI/HMA/VEN cohort and lowest in the DLI/HMA cohort (Table 2; Fig. 2). Duration and type of concomitant treatments were heterogeneous and are shown in Fig. 1: Patients in the DLI/HMA cohort received a median of 1 (range: 1–9) cycles of HMA. Patients in the DLI/HMA/VEN cohort received a median of 4 (range: 1–20) cycles of HMA+/-VEN in total, consisting of a median of 2 cycles (range: 1–8) of HMA/VEN combination therapy and a median of another 1 cycle (range: 0–14) of HMA treatment without VEN administration. Patients in the other/no treatment cohort received up to 17 cycles of their respective accompanying treatments.

The median follow-up of our study was 43.7 months (range: 4.7-224.3 months) after alloHCT and was longest in the DLI/HMA cohort and shortest in the DLI/other or no treatment cohort (DLI/HMA: 56.5 (5.1-224.3), DLI/HMA/VEN: 42.2 (7.6-188.8), DLI/other or no treatment: 37.8 (4.7-194.1) months).

### Outcome after DLI treatment

**Fig. 3:**
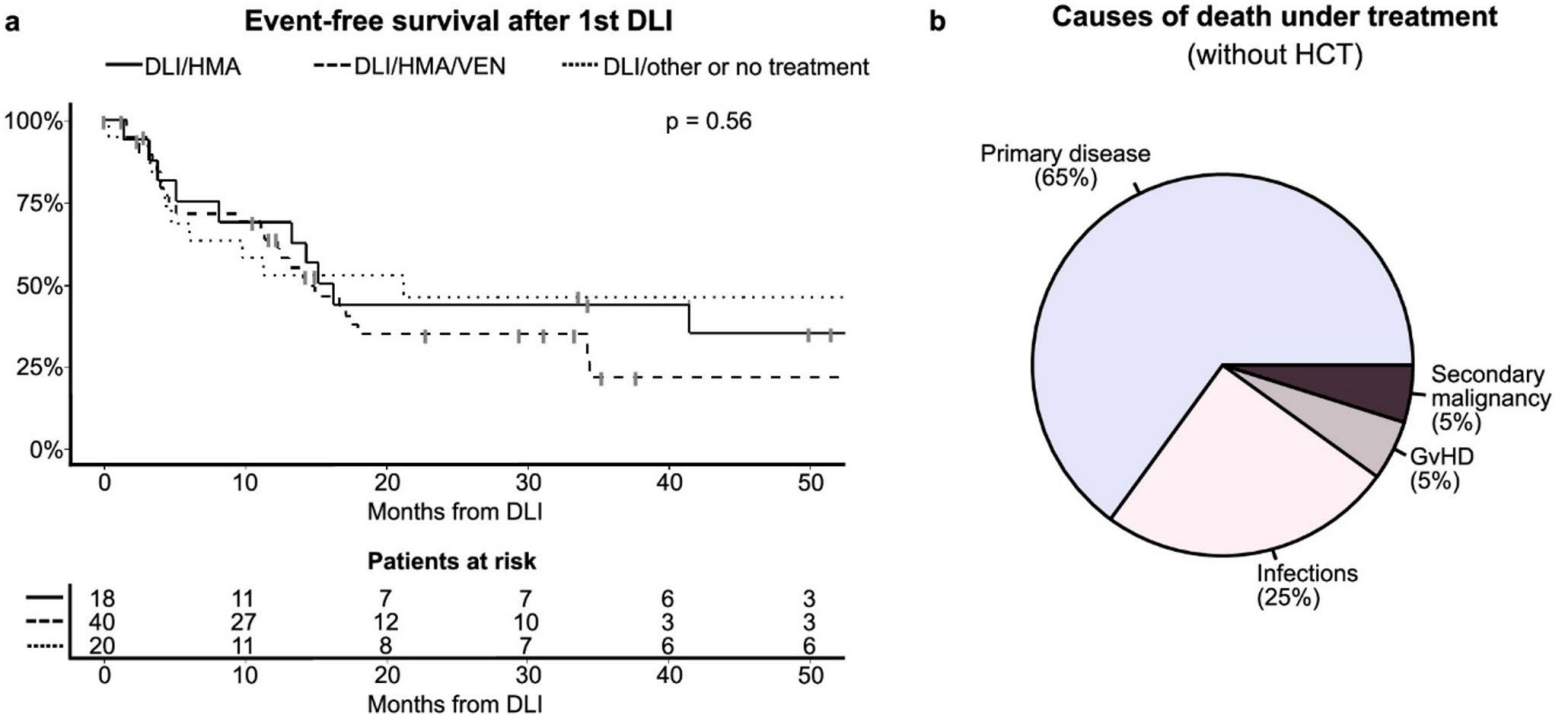
Outcome after DLI treatment. **a** Kaplan Meier curves show EFS after first DLI for the different treatment groups: patients receiving DLI/HMA (continuous line, *n*=18), patients receiving DLI/HMA/VEN (dashed line, *n*=40) and patients receiving DLI/other or no treatment (dotted line, *n*=20). **b** Causes of death in 20 patients under treatment (without consecutive HCT). Causes of death include relapse or progress of primary disease, secondary cancer, GvHD and infections. DLI, donor lymphocyte infusion; EFS, event-free survival; HMA, hypomethylating agent; VEN, venetoclax; GvHD, graft versus host disease

Median EFS after first DLI was 15.2 months in the entire cohort, 16.2 months in the DLI/HMA group, 14.3 months in the DLI/HMA/VEN group and 21.2 months in the DLI/other or no treatment group, respectively (Fig. 3A). EFS was comparable between patients who received above and below average cumulative T-cell count/kg bodyweight (Supplementary Fig. 2) but significantly lower in patients experiencing hematological relapse (*p* = 0.001, Supplementary Fig. 3).

Events contributing to EFS included second alloHCT in 32.1% (*n* = 25) and death without second alloHCT in 25.6% (*n* = 20/78) of patients. Patients from the DLI/other or no treatment group were less likely to receive a second alloHCT (20.0%) than patients treated with DLI/HMA (33.3%) or DLI/HMA/VEN (37.5%, Table 1). Among the 20 patients who died without proceeding to second alloHCT (DLI/HMA: *n* = 3/18, 16.7%; DLI/HMA/VEN: *n* = 11/40, 27.5%; DLI/other or no treatment: *n* = 6/20, 30.0%; Fig. 3B), the leading cause of death was relapse or progressive disease, accounting for 65% of deaths, followed by infections (25%). One patient (5%) died from DLI-triggered GvHD and one patient (5%) died from secondary malignancy (NSCLC) (Fig. 3B).

The 2-year-OS of all relapsed patients treated with DLI after first alloHCT, including patients proceeding to receive secondary alloHCT, was 73.7% in the entire cohort, 76.5% in the DLI/HMA group, 74.7% in the DLI/HMA/VEN group and 63.7% in the DLI/other or no treatment group, respectively (Supplementary Fig. 5). Note that an additional 21 patients (26.9%) who proceeded to second alloHCT subsequently died; these deaths are included in the OS analysis but were excluded from the causes of death analysis described above.

Graft versus Host Disease (GvHD) was triggered in 30.8% of patients after DLI (acute: 17.9%, chronic: 16.7%) and was graded mostly as acute grade I-II or moderate cGvHD (Table 2). Overall GvHD-rates after DLI did not differ significantly between cohorts (Table 2).

Lymphocyte counts as well as T-cell counts in peripheral blood after relapse were largely comparable between the cohorts (Supplementary Fig. 6A-B).

In-house documented infectious adverse events, as classified by the common terminology criteria for adverse events (CTCAE), were comparable between the cohorts with a mean of 0.8 infectious episodes during treatment per patient (Supplementary Fig. 4A). Most infections were grade III. In the DLI/HMA/VEN cohort, six grade IV-V infections were documented (Supplementary Fig. 4B-D).

Subgroup analysis: complete responders and AML with *TP53* mutations.

CR was achieved in 42.3% of patients (*n* = 32) after DLI treatment. For seven patients (9.0%), remission status was not available due to patients’ demise prior to response evaluation. Median time from DLI to CR was 4.2 (range: 0.7–14.7) months in the entire cohort (DLI/HMA: 5.3 (range: 2.2–11.8), DLI/HMA/VEN: 3.3 (1.7–12.0), DLI/other or no treatment: 4.2 (0.7–14.7) months). CR rates were slightly higher in the DLI/other or no treatment cohort (50.0% vs. 33.3% in DLI/HMA and 42.5% in DLI/HMA/VEN, Table 2). Concomitant mutations at primary diagnosis of patients achieving CR under treatment are presented in Supplementary Fig. 7A. Complex karyotype at primary diagnosis was prevalent in 15 patients achieving CR, with those patients being almost exclusively included in the DLI/HMA/VEN cohort (DLI/HMA/VEN: 12 patients, DLI/HMA: 2 patients, DLI/other or no treatment: 1 patient). Among patients achieving CR, nine cases (27.3%) with *TP53* mutations were included (entire cohort: 28 cases (35.9%) with *TP53* mutations). Exploratory analysis of AML patients with TP53 mutations showed a median OS of 66.9 months compared to 93 months in AML patients without *TP53* mutation, and median EFS of 11.4 months compared to 17.1 months in patients without *TP53* mutation; however, these differences did not reach statistical significance (Supplementary Fig. 7).

## Discussion

Since its first proof of principle in 1990 [35], DLI have been an established treatment after alloHCT and have been widely applied in most centers [8]. DLI are being used for both mixed chimerism, preemptively or in both molecular or hematological relapse [8]. Despite its wide use recommendations on DLI treatment rely on expert opinions and retrospective evidence [7] as DLI treatment is difficult to standardize. This is due to a high number of confounding factors e.g. degree of HLA-match between recipient and donor, timepoint of administration (early or late after alloHCT), with concurrent immunosuppression or without, immunogenicity or immunomodulating capacity of underlying disease, occurrence of GvHD, T-cell count within the DLI as well as availability of DLI aliquots. The same limitations need to be taken into consideration when interpreting our retrospective analysis of current center practice.

Diverging from consensus recommendations for DLI dosing, suggesting between 1 and 5 × 10^6^ CD3^+^ cells/kgbw as treatment for molecular relapse and 10 × 10^6^ CD3^+^ cells/kgbw in case of hematological relapse, initial DLI graft cell counts in our cohort started ad as little as 0.3-x10^6^ CD3^+^ cells/kgbw but also included single cases of first dosages of 8.14 × 10^6^ CD3^+^ cells/kgbw [7, 36]. Step up dosing varied largely between the cohorts and was fastest in the DLI/HMA/VEN cohort (Fig. 2). In the context of moderate overall GvHD rates and the relatively small amount of severe cGvHD cases following DLI treatment (Table 2), despite our conservative initial dosing strategy and achievement of substantial cumulative DLI doses, our data suggest that GvHD risk may remain manageable even with dose escalation. Administering preemptive DLI dosages prior to MRD conversion has been reported to prevent relapse after alloHCT and may offer an additional approach for disease-control in these high-risk populations [6, 37].

While DLI/HMA/VEN patients received overall higher DLI dosages and a distinctively higher mean cumulative DLI graft count (Fig. 2), in median only two (range 1–8) cycles of complete DLI/HMA/VEN combination treatment were administered (Table 2, Fig. 1). Main reasons for de-escalation of therapy were aggravated cytopenia and infectious complications under medication with VEN (Supplementary Fig. 4), as well as achievement of remission, after which treatment was continued with less toxic regimens (HMA + DLI or DLI monotherapy). Similarly, in the DLI/HMA cohort, HMA was typically discontinued upon achieving remission. Importantly, unlike continuous targeted therapies such as TKIs, our approach combines time-limited chemotherapy with ongoing immunological pressure from DLI, which may contribute to durable responses despite relatively brief pharmacological treatment courses. Our findings are in line with other studies, reporting HMA/VEN treatment in post-alloHCT as well as non-alloHCT-settings [38, 39].

We set out to address the question if the addition of venetoclax to the portfolio of recurrence treatments for myeloid malignancies also improves outcome after alloHCT. While the combination of HMA/VEN has revolutionized treatment of refractory AML in pre-alloHCT settings [25, 40–42], our data showed no clear advantage in EFS or OS for HMA/VEN in the context of DLI treatment post-alloHCT (Fig. 3A and Supplementary Fig. 5). This might partially be due to a biased cohort composition with higher risk patients and more hematological relapses in the DLI/HMA/VEN cohort (Tables 1 and 2). Adverse risk, relapsed/refractory disease as well as hematological relapse post alloHCT are linked to higher mortality rates after alloHCT, thus affecting treatment decisions and introducing a selection bias [28, 43–45]. Hypothetically, venetoclax itself could impair the GvL effect by exerting immunosuppressive or immunomodulatory effects or increasing infectious morbidity and mortality, a hypothesis that we could not find evidence for in an explorative analysis of lymphocyte counts or infectious episodes of our cohort. Lymphocyte and T-cell counts as well as GvHD rates were largely comparable between all three cohorts (Supplementary Fig. 6, Fig. 3A; Table 2).

Whilst we could not demonstrate superiority of HMA/VEN/DLI over HMA/DLI and DLI/no other treatment, our results show approximately 40% CR rates after DLI among the whole cohort with an approximately 70% 2-year OS after diagnosis of a relapsed malignancy after alloHCT (including 2nd transplant). Notably, responses were also present in relapsed cases with *TP53* mutations though the limited sample size and lack of detailed variant allele frequency and cytogenetic data preclude definitive conclusions regarding outcomes in this high-risk subgroup. These findings are consistent with other recent works, where presence of *TP53*-mutations alone did not impact survival until stratified for mutational burden or transplant type and MRD status [46–48], suggesting that additional molecular and cytogenetic characterization is necessary to fully understand the prognostic impact of *TP53* mutations in the post-transplant relapse setting.

In conclusion, we were able to show that DLI combination therapies represent a safe and efficient therapy to either salvage or bridge patients with myeloid neoplasms to second transplant with a manageable safety profile. Observing that severe GvHD complications were manageable in all cohorts, our data suggests that a brisker deployment of larger DLI grafts in case of relapsed myeloid neoplasms after alloHCT can be considered. Notably, the DLI/HMA/VEN cohort included a higher proportion of patients with AML and morphological relapse, traditionally higher-risk features, yet achieved comparable outcomes to the other treatment groups. This suggests that HMA/VEN combination therapy may successfully salvage even morphologically relapsed disease when combined with DLI. However, given the baseline differences between cohorts and the non-randomized nature of this study, we cannot definitively determine the incremental benefit of venetoclax addition. A prospective, ideally randomized trial would be required to formally assess whether the addition of venetoclax to DLI-based salvage therapy improves outcomes compared to DLI with HMA alone or other treatment approaches.

## Supplementary Information

Below is the link to the electronic supplementary material.

Supplementary Material 1.

## Data Availability

Pseudonymised data as well as written R code are available from the authors upon reasonable request.
