# Supplementary material for: Donor lymphocyte infusions for recurrence of myeloid neoplasms after allogeneic hematopoietic cell transplantation in the era of hypomethylating agents and BCL2 inhibitors
Source: Ann Hematol. 2026 Mar 30;105(4):213. doi: 10.1007/s00277-026-06973-7 (PMC13035613; doi:10.1007/s00277-026-06973-7)
Supplement: Supplementary file 1 — Supplementary Material 1. [file 277_2026_6973_MOESM1_ESM.pdf]

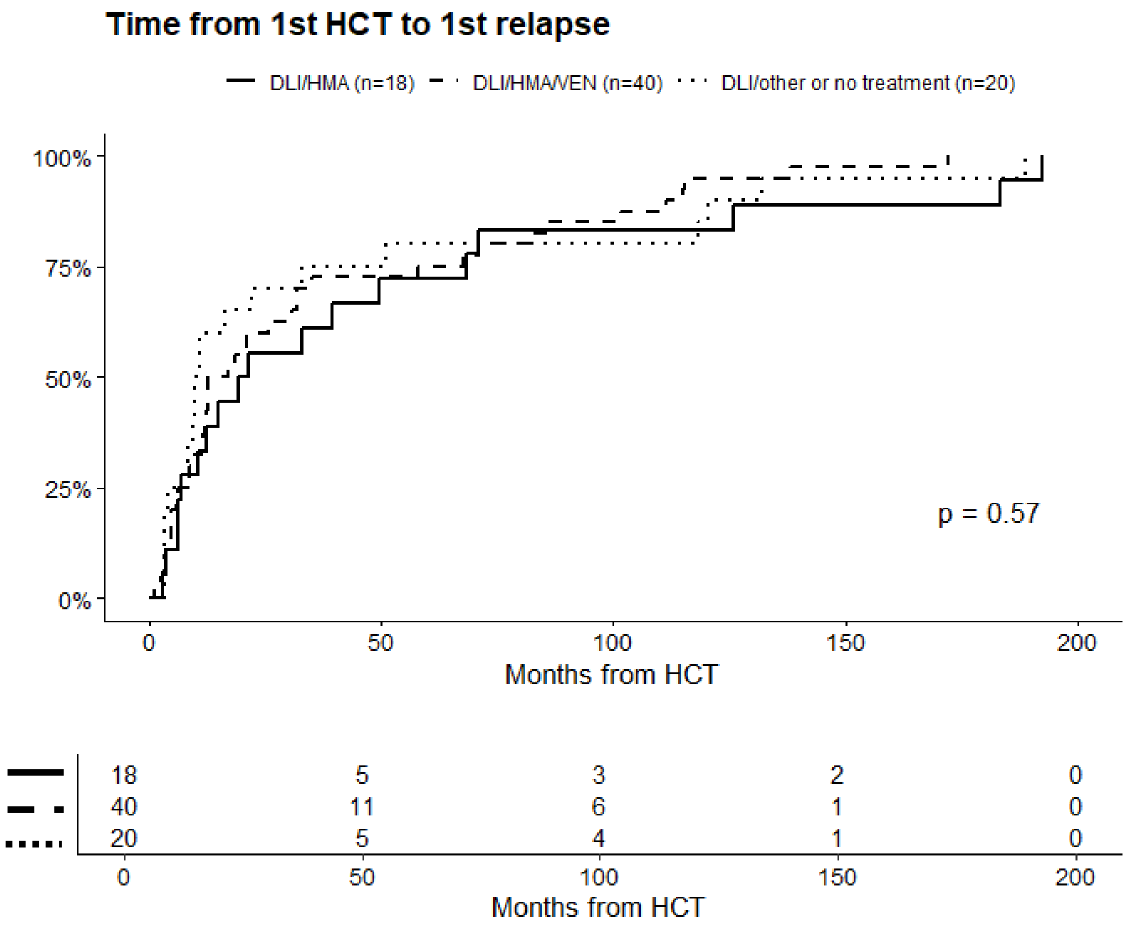

Supplementary Figure 1: Time to first relapse after HCT.
Kaplan Meier Curves show the time from first HCT to first relapse of any kind (hematological or molecular) for the different treatment groups: patients receiving DLI/HMA (continuous line, n=18), patients receiving DLI/HMA/VEN (dashed line, n=40) and patients receiving DLI/other or no treatment (dotted line, n=20).
HCT, hematologic cell transplantation; HMA, hypomethylating agent; DLI, donor lymphocyte infusion.

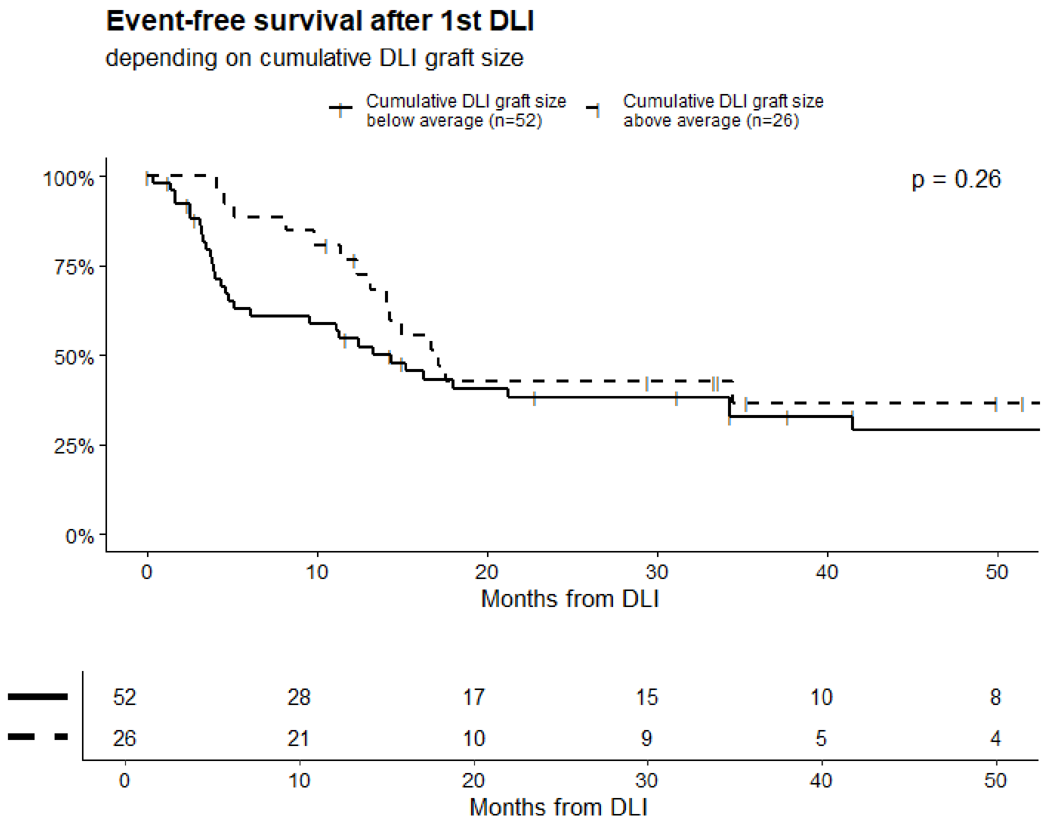

Supplementary Figure 2: EFS after first DLI for patients receiving above or below average cumulative DLI graft size. Kaplan Meier Curves show EFS after first DLI split by cumulative DLI graft size: patients receiving below average cumulative DLI graft size (continuous line, n=52) and patients receiving above average cumulative DLI graft size (dashed line, n=26).

EFS, event-free survival; DLI, donor lymphocyte infusion.

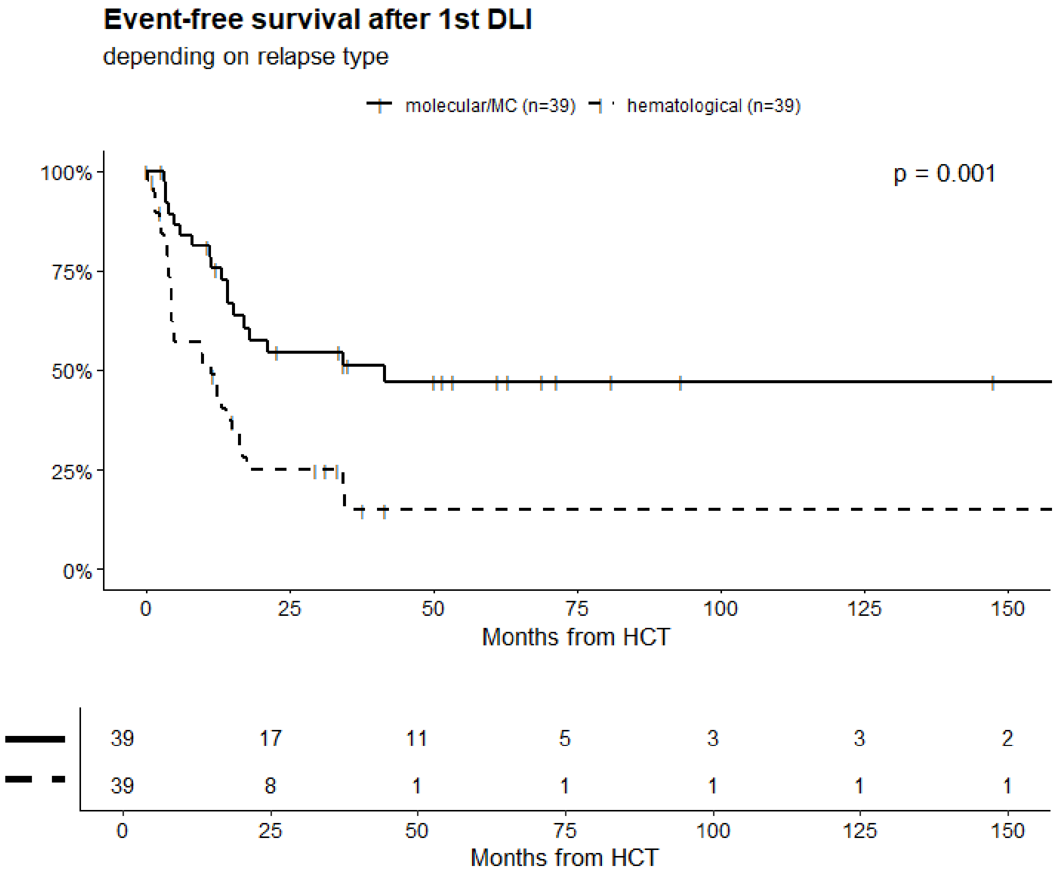

Supplementary Figure 3: EFS after first DLI depending on type of relapse.

Kaplan Meier Curves show EFS after first DLI depending on type of relapse after alloHCT: patients experiencing molecular relapse or mixed chimerism prior to receiving DLI (continuous line, n=39) and patients experiencing hematological relapse prior to receiving DLI (dashed line, n=39).

EFS, event-free survival; DLI, donor lymphocyte infusion, MC, mixed chimerism, HCT, hematologic cell transplantation.

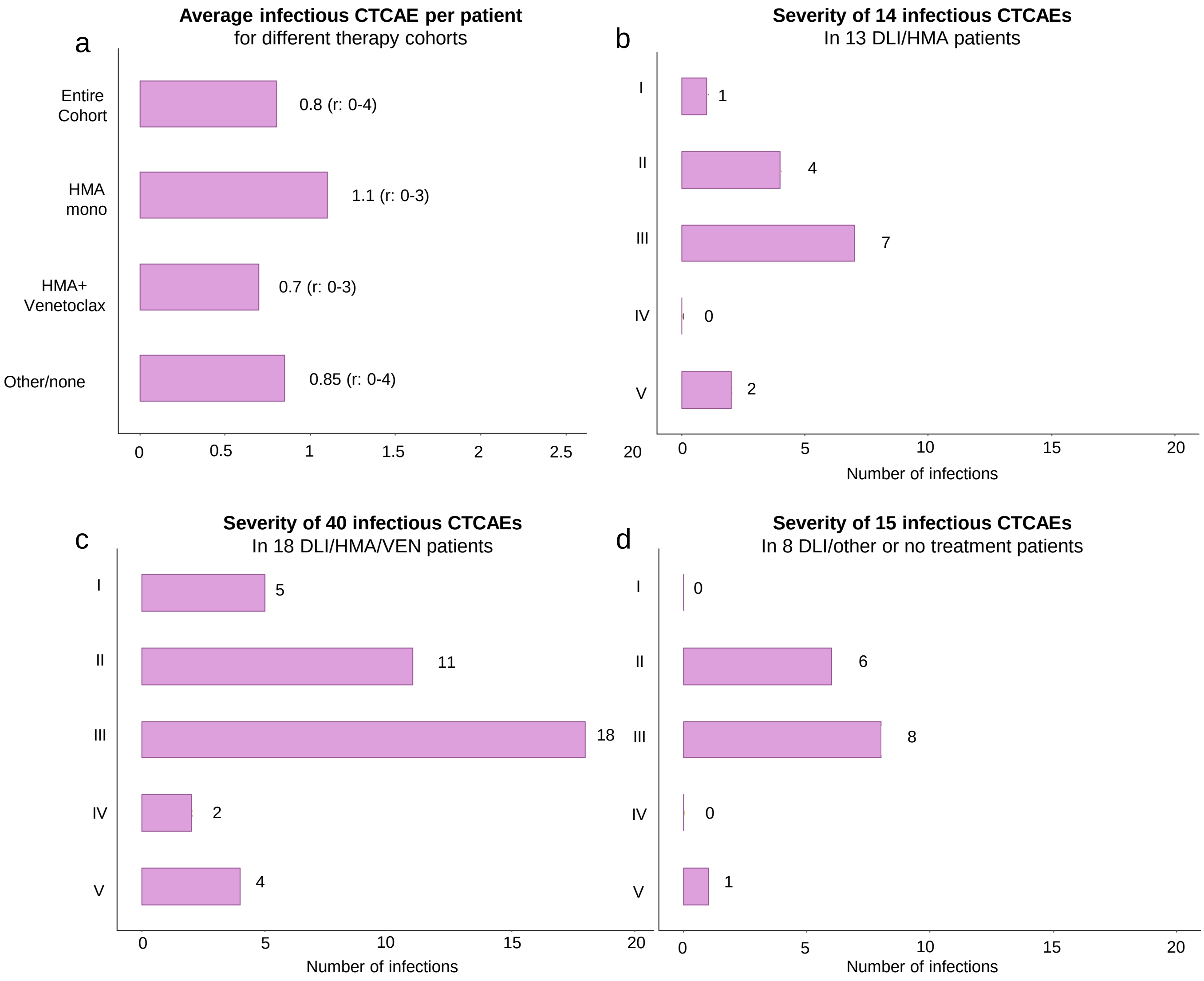

Supplementary Figure 4: Infectious CTCAE.
a. Average prevalence of infectious CTCAE per patient in the entire cohort.
b. Severity of CTCAE in 14 cases in 13 DLI/HMA patients.
c. Severity of CTCAE in 40 cases in 18 DLI/HMA/VEN patients.
d. Severity of CTCAE in 15 cases in 8 DLI/other or no treatment patients.
DLI, donor lymphocyte infusion; HMA, hypomethylating agent; CTCAE, common terminology criteria for adverse events.

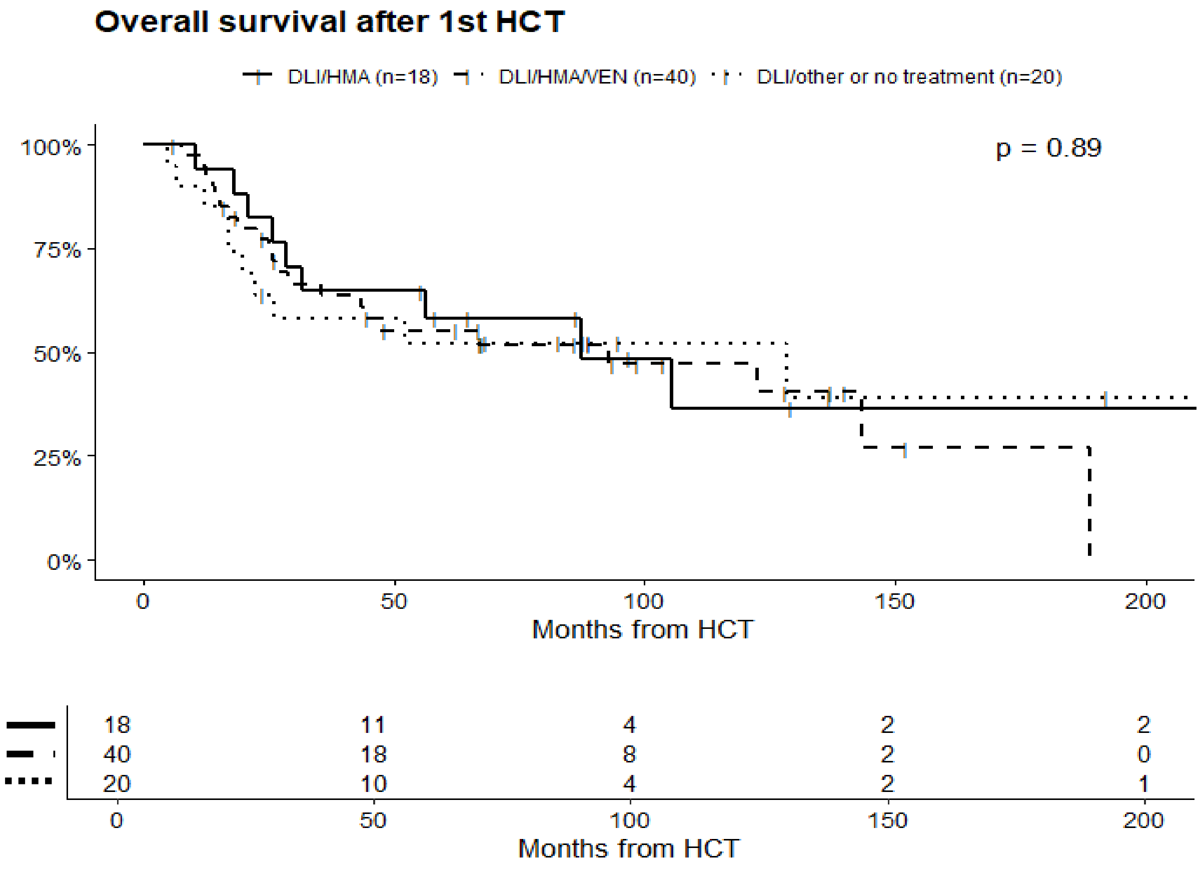

Supplementary Figure 5: OS after HCT.

Kaplan Meier Curves show OS after HCT in patients experiencing relapse and treated with DLI for the different treatment groups: patients receiving DLI/HMA (continuous line, n=18), patients receiving DLI/HMA/VEN (dashed line, n=40) and patients receiving DLI/other or no treatment (dotted line, n=20).

OS, overall survival; HCT, hematologic cell transplantation; HMA, hypomethylating agent; DLI, donor lymphocyte infusion.

a

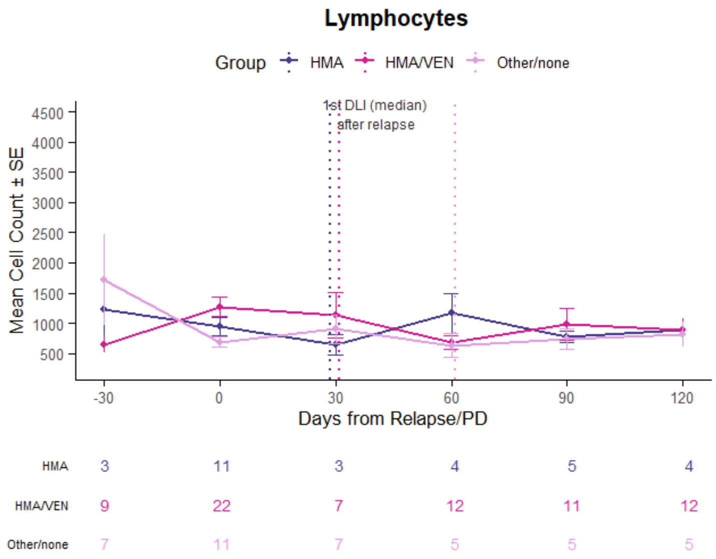

b

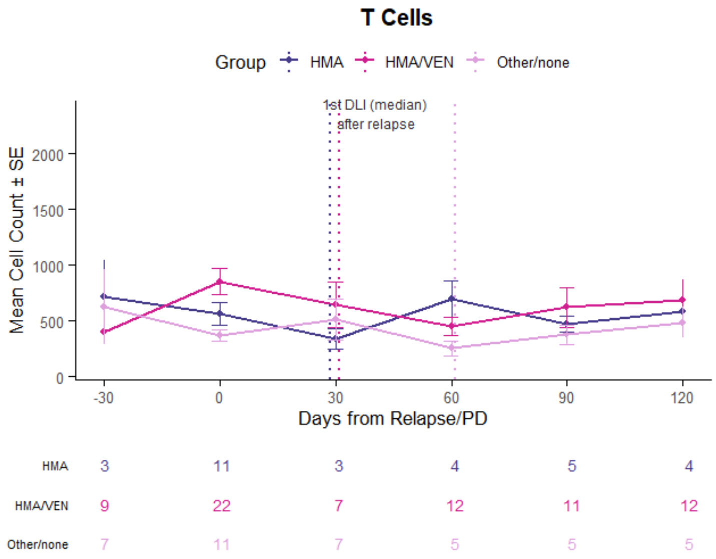

Supplementary Figure 6: Cell Counts after Relapse.
The panels show the mean cell counts in peripheral blood and error margins for the different cohorts at several timepoints preceding and following relapse or progressive disease after allogeneic stem cell transplantation. The DLI/HMA cohort (n=18) is depicted by the dark blue line, the DLI/HMA/VEN cohort (n=40) is depicted by the dark violet line and the DLI/other or no treatment cohort (n=20) is depicted by the light violet line. Availability of data sets vary between timepoints.
Panel **a** shows total lymphocyte count, panel **b** shows total T-cell count.
DLI, donor lymphocyte infusion; HMA, hypomethylating agent; VEN, venetoclax; NK-cells, natural killer cells; CR, complete remission; PD, progressive disease.

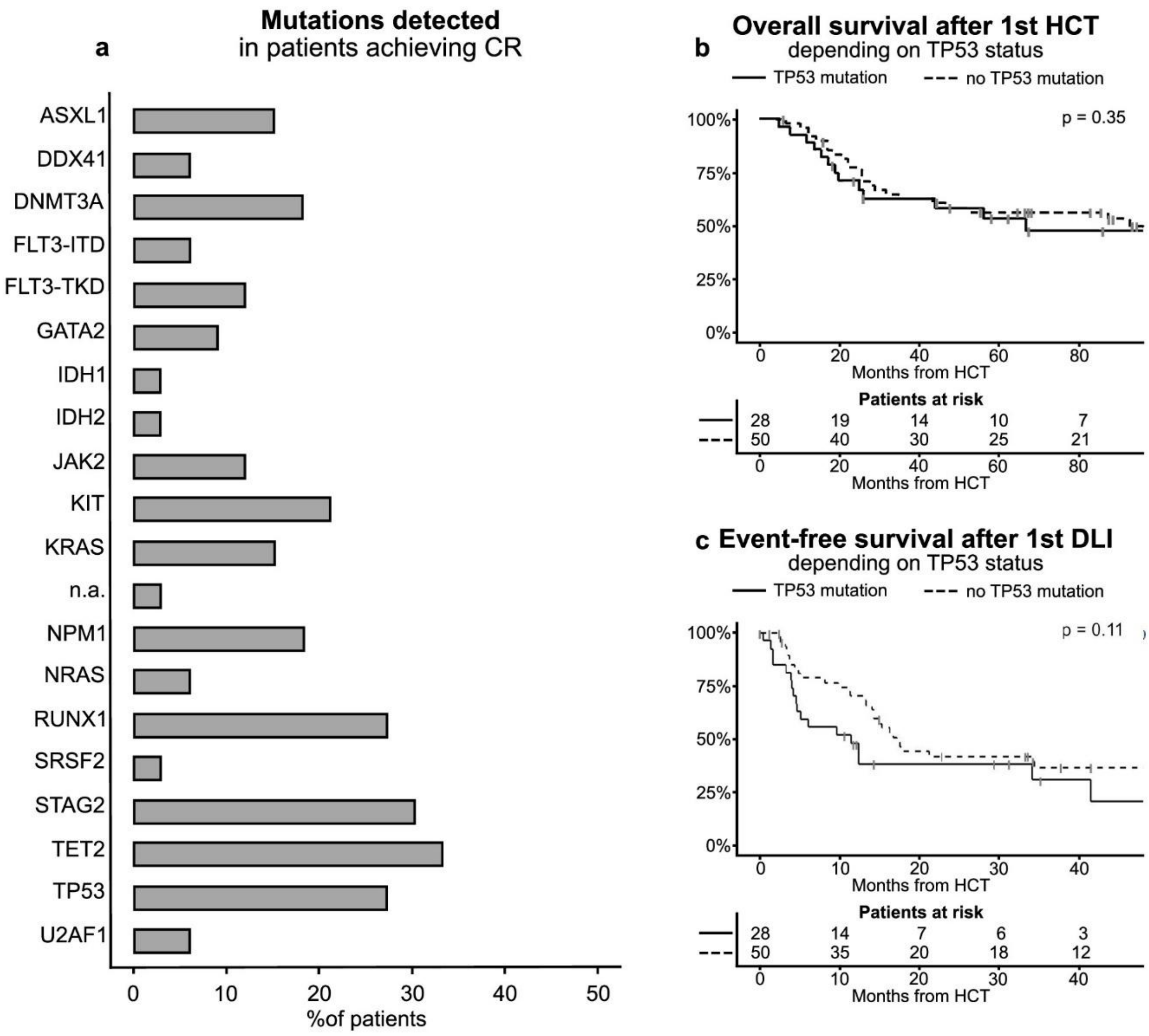

Supplementary Figure 7: Mutation analysis of patients achieving CR.

a. The Bar plots show concomitant mutational patterns in patients achieving CR after relapse for the entire cohort.

b. Kaplan Meier curves show OS after first HCT for patients with TP53 mutations (continuous line, n=28) and without TP53 mutations (dashed line, n=50).

c. Kaplan Meier curves show EFS after first DLI for patients with TP53 mutations (continuous line, n=28) and without TP53 mutations (dashed line, n=50).

DLI, donor lymphocyte infusion; EFS, event-free survival; OS, overall survival; HMA, hypomethylating agent; VEN, venetoclax.
